# Psychogenic Facial Movement Disorders: Clinical Features and Associated Conditions

**DOI:** 10.1002/mds.25190

**Published:** 2012-10-02

**Authors:** Alfonso Fasano, Anabela Valadas, Kailash P Bhatia, LK Prashanth, Anthony E Lang, Renato P Munhoz, Francesca Morgante, Daniel Tarsy, Andrew P Duker, Paolo Girlanda, Anna Rita Bentivoglio, Alberto J Espay

**Affiliations:** 1 Dipartimento di Neuroscience, Università Cattolica del Sacro CuoreRome, Italy; 2 Department of Neuroscience AFaR-Fatebenefratelli Association for Biomedical Research “San Giovanni Calibita-Fatebenefratelli” HospitalIsola Tiberina, Rome; 3 Department of Neurology, Hospital de Santa Maria, Centro Hospitalar Lisboa NorteEPE, Lisbon, Portugal; 4 Sobell Department of Motor Neuroscience and Movement Disorders, UCL, Institute of Neurology, University College LondonUnited Kingdom; 5 Division of Neurology, Toronto Western Hospital, University of TorontoToronto, Ontario, Canada; 6 Service of Neurology, Pontifical Catholic University of ParanaCuritiba, Brazil; 7 Dipartimento di Neuroscienze, Scienze Psichiatriche ed Anestesiologiche, Università di MessinaMessina, Italy; 8 Beth Israel Deaconess Medical Center, Harvard Medical SchoolBoston, Massachusetts, USA; 9 UC Neuroscience Institute, Department of Neurology, Gardner Center for Parkinson's Disease and Movement Disorders, University of CincinnatiCincinnati, Ohio, USA

**Keywords:** facial movement disorders, psychogenic movement disorders, psychogenic facial movement disorders, psychogenic dystonia, psychogenic blepharospasm, facial distortion

## Abstract

The facial phenotype of psychogenic movement disorders has not been fully characterized. Seven tertiary-referral movement disorders centers using a standardized data collection on a computerized database performed a retrospective chart review of psychogenic movement disorders involving the face. Patients with organic forms of facial dystonia or any medical or neurological disorder known to affect facial muscles were excluded. Sixty-one patients fulfilled the inclusion criteria for psychogenic facial movement disorders (91.8% females; age: 37.0 ± 11.3 years). Phasic or tonic muscular spasms resembling dystonia were documented in all patients most commonly involving the lips (60.7%), followed by eyelids (50.8%), perinasal region (16.4%), and forehead (9.8%). The most common pattern consisted of tonic, sustained, lateral, and/or downward protrusion of one side of the lower lip with ipsilateral jaw deviation (84.3%). Ipsi- or contralateral blepharospasm and excessive platysma contraction occurred in isolation or combined with fixed lip dystonia (60.7%). Spasms were reported as painful in 24.6% of cases. Symptom onset was abrupt in most cases (80.3%), with at least 1 precipitating psychological stress or trauma identified in 57.4%. Associated body regions involved included upper limbs (29.5%), neck (16.4%), lower limbs (16.4%), and trunk (4.9%). There were fluctuations in severity and spontaneous exacerbations and remissions (60%). Prevalent comorbidities included depression (38.0%) and tension headache (26.4%). Fixed jaw and/or lip deviation is a characteristic pattern of psychogenic facial movement disorders, occurring in isolation or in combination with other psychogenic movement disorders or other psychogenic features. © 2012 Movement Disorder Society

Many systemic and neurological conditions may involve the facial musculature. From tetanus to blepharospasm, the majority of them are characterized by muscular spasms.^1^ While some of them are easily recognizable, anecdotal reports have recently focused attention on atypical presentations.^2^–^5^ Although most of these cases have been reported as representing rare phenotypes of organic focal dystonia,^3^–^5^ these patients may fulfill diagnostic criteria for psychogenic movement disorders (PMDs).

PMDs involving the face have been largely described as blepharospasm, reported in 3%^6^ to 7%^7^ of all types of PMD, and in 22% of a consecutive series of 50 patients in a botulinum toxin injection clinic.^8^ However, the features and diagnostic clues of the wider range of psychogenic facial movement disorders (PFMDs), as well as the relationship with previously reported cases in the *Movement Disorders* journal^3^ and others,^1^ remain unknown. We sought to examine a large series of PMDs where the orofacial region was involved in order to determine the clinical features and associated disorders, and to highlight their inconsistency and incongruence with recognized organic movement disorders.

## Patients and Methods

Seven tertiary movement disorders centers performed a retrospective chart review of PMD involving the face using computerized databases and, when available, videotape examinations of patients evaluated between January 1993 and January 2010. Only patients actively followed up and treated by experts in movement disorders were included. Diagnosis of PMD was made according to the criteria of Fahn and Williams^6^ and Gupta and Lang.^9^ Patients were excluded when their clinical features were in keeping with those of hemimasticatory spasm or geniospasm or when they fulfilled established diagnostic criteria for dystonia (blepharospasm, oromandibular dystonia, Meige syndrome, or other dystonias, either focal or in the context of segmental or generalized forms), hemifacial spasm, or any neurological disease known to affect facial muscles (eg, myasthenia gravis, Parkinson's disease, chorea, epilepsy). The following data were collected using a standardized spreadsheet: familial and demographic data, clinical history, extent and type of facial and extra-facial involvement, neurophysiological investigations, type and outcome of the treatments performed by the movement disorders specialist (including psychotherapy and related techniques administered by other physicians upon request of the former) and, when available, long-term outcome data. Associated comorbidities and psychogenic features (including somatizations, false weakness or sensory complaints, suggestibility, or deliberate slowness of movements), secondary gain (ongoing or pending litigation, disability benefits, release from personal/legal/social/employment responsibilities, and/or increased personal attention), and disproportionate functional disability were also documented. Psychiatric diagnoses followed the Diagnostic and Statistical Manual of Mental Disorders (DSM-IV TR) criteria.^10^ The diagnosis of a PFMD was not made on the basis of the presence or nature of associated psychiatric disturbances but on the clinical assessment of experienced movement disorders neurologists whose observations form the basis of this report.

## Results

### Demographics

The seven centers retrieved a total of 87 patients with PFMDs, representing the 16.3% ± 15.8% of all the PMDs cases seen during the examined period (range: 5.0% [Rome] to 50.0% [Boston]). Twenty-six cases were excluded because of missing data (eg, no video or limited follow-up) or uncertain clinical features, thus resulting in a total of 61 patients with PFMDs (92% females; mean age at onset, 37 ± 11.3 years; disease duration 6.7 ± 6.9 years; Table 1). They met published diagnostic criteria for PMDs (DSM-IV TR criteria for conversion disorder, 40.7%; somatization disorder, 9.8%; malingering, 4.9%).

**TABLE 1:** Demographic and clinical features, categories of diagnostic certainty, and instrumental investigations undergone by the patients fulfilling the inclusion criteria for PFMDs

N	61
Females (%)	56 (91.8%)
Educational level (y)	11.3 ± 3.7 (7–17)
Marital status	
Married	38 (62.3%)
Single	15 (24.6%)
Divorced	6 (9.8%)
Unmarried partner	1 (1.6%)
Widowed	1 (1.6%)
Disease duration (y)	6.7 ± 6.9 (0–30)
Age (y)	43.7 ± 11.5 (19–66)
Follow-up duration (mo)	40.8 ± 46.6 (0–228)
Number of visits	4.0 ± 4.1 (1–29)
Family history of neurological disorders^a^	2 (3.3%)
Exposure to neuroleptics^b^	1 (1.6%)
Treatment with antidepressant prior to PFMD onset	14 (22.9%)
Treatment with benzodiazepines prior to PFMD onset	6 (9.8%)
Precipitating events	
Psychological stress	22
Physical trauma	9
Peripheral facial injury	8
Pain	3
Another disease	5
Site of symptom onset	
Face (n = 56)	
Lips:	37 (60.7%)
Eyelids	23 (37.7%; concurrently with lips in 7)
Forehead	2 (concurrently with lips in 1)
Platysma muscle	2
Outside face (n = 5)	3
Cervical muscles	
Abdominal muscles	1
Foot	1
Onset	
Abrupt	49 (80.3%)
Subacute^c^	10 (16.4%)
Gradual	2 (3.3%)
Diagnostic certainty (Fahn and Williams criteria^6^)	
Documented	13 (21.3%)
Clinically established	43 (70.5%)
Probable	5 (8.2%)
Diagnostic certainty (Gupta and Lang criteria^9^)	
Documented	13 (21.3%)
Clinically established plus other features	39 (63.9%)
Clinically established minus other features	9 (14.8%)
Disclosure of diagnosis of psychogenicity to patient	41 (67.2%)
Neuroimaging^d^ (n = 57)	Normal in all but 3 patients (mild cortical atrophic changes or nonspecific white matter abnormalities)
MRI angiography (n = 7)	Vascular loop compressing the root of facial nerve in 1 patient
VEP (n = 5)	Normal
BAEP (n = 5)	Normal
SEP (n = 5)	Normal
MEP (n = 5)	Normal
EEG (n = 9)	Normal
EMG (n = 21)	Normal
Blink reflex (n = 10)	Normal
CSF analysis (n = 10)	Normal

Values are mean ± SD (range).

a One patient with normal neuroimaging had a family history of Fahr's disease and other a daughter with unspecified ataxia.

b Taken for few weeks and several years before PFMD onset.

c Subacute onset for cases reaching the greatest severity in 1 month.

d MRI in all but 3, who underwent computed tomography. PFMD, psychogenic facial movement disorder; MRI, magnetic resonance imaging; VEP, visual evoked evoked potentials; BAEP, brainstem auditory evoked potentials; SEP, somatosensory evoked potentials; MEP, motor evoked potentials; EEG, electroencephalogram; EMG, electromyogram; CSF, cerebrospinal fluid.

### Clinical Features

**FIG. 1:**
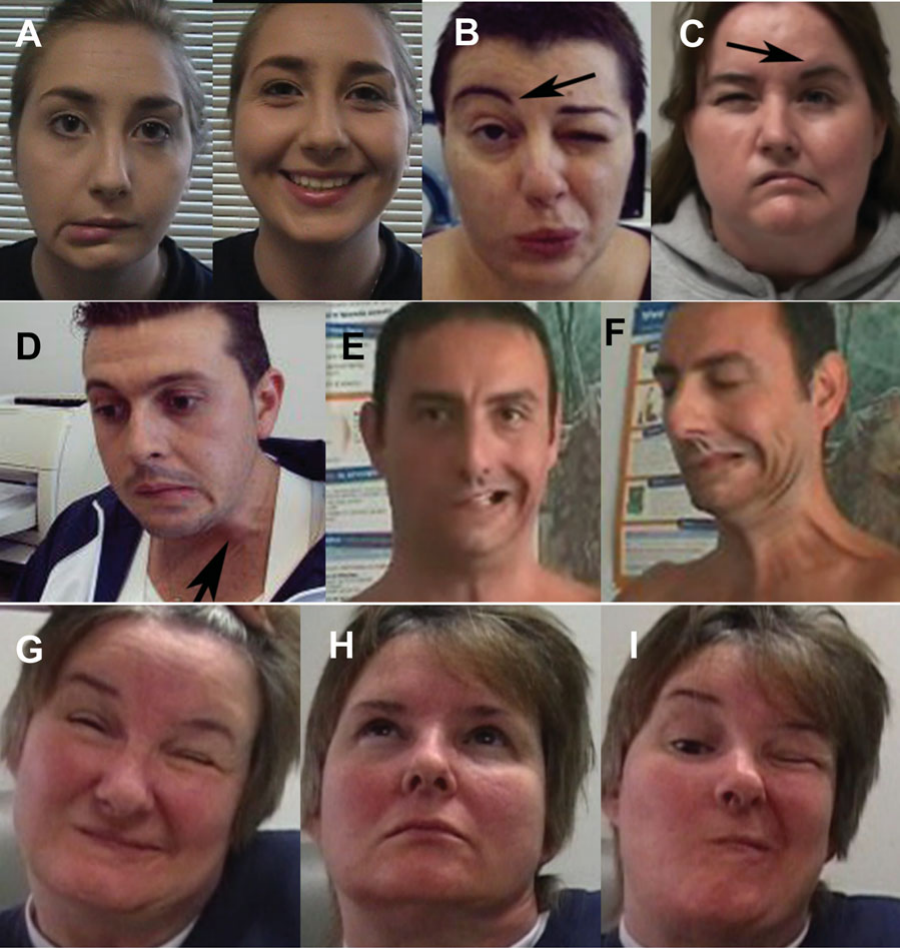
The clinical presentation of PFMDs. The most common phenotype, isolated lower lip dystonia (**A**), in a patient with spontaneous remissions and intermittent ipsilateral jaw deviation (see also Video 1); eyelid spasm may be ipsilateral (**B**) or contralateral to the lip pulling (**C**) (see also Video 2). Note that the contraction of frontalis muscle involves the eyebrow contralateral to the spasm of the orbicularis oculi (arrows indicates a false “other Babinski sign”); platysma involvement is always associated with ipsilateral lip involvement (**D**), which can rapidly fluctuate in severity and appearance (**E, F**, same case); Some patients demonstrated severe bilateral spasms of most facial musculature (**G**), which may remit after placebo (vibrating tuning fork application, **H**) and relapse with different phenomenology shortly thereafter (**I**).

All patients had phasic or tonic muscular contractions resembling dystonia. The most prevalent feature was lip pulling (83.6%), predominantly downward (Fig. 1). Sixty percent had paroxysmal or intermittent symptoms; fixed posturing was noted in most of the remainder. No patients acknowledged voluntary control or premonitory urge. Unilateral involvement was documented in most (84.3%), with alternating sides only in 2 subjects. There was lip pain and associated excessive ipsilateral platysma contractions in two-thirds of these. Other clinical features are listed in Table 2.

**TABLE 2:** The clinical features of patients with PFMDs with lip involvement

N	51
Involvement of	
Any other facial muscle	24 (47.1%)
Platysma	37 (72.5%)
Neck, trunk, or limbs	22 (43.1%)
Onset outside face	5 (9.0%)
Facial/head pain	19 (37.2%)
Paroxysmal or action-induced only at onset	9 (17.6%)
Action-induced	3 (5.9%)
Paroxysmal symptoms	29 (56.9%)
Dystonic fixed posture	14 (27.5%)
Dystonic movement	40 (78.4%)
Consistency of laterality	40 (78.4%)
Asymmetry	43 (84.3%)
Side	
Right	16 (31.4%)
Left	20 (39.2%)
Both asynchronous	7 (13.7%)
Both synchronous	8 (15.7%)
Direction of lip pulling	
Upward	13 (25.5%)
Downward	32 (62.7%)
Both directions	3 (5.9%)
Sideways	3 (5.9%)
Type of speech	
Normal	26/46 (56.5%)
Slurred	11/46 (23.9%)
Burst of verbal gibberish	7/46 (15.2%)
Stuttering	2/46 (4.3%)
Geste antagoniste (eg, placing in mouth a cigarette or a pencil)	3/49 (6.1%)
Effect of speech	
No effect	28 (54.9%)
Improvement	8 (15.7%)
Worsening	15 (29.4%)
Effect of eating	
No effect	33/41 (80.5%)
Improvement	6/41 (14.6%)
Worsening	2/41 (4.9%)
Effect of mouth movements (eg, kissing, whistling)	
No effect	7/20 (35.0%)
Improvement	8/20 (40.0%)
Worsening	5/20 (25.0%)
Resolution during sleep	17/21 (80.9%)

PFMD, psychogenic facial movement disorder.

Paroxysmal (65%) or fixed (26%) eyelid involvement occurred mostly unilateral with alternating sides (65%). The right side was affected twice as often as the left (11 vs 5). Three cases reported gestes antagonistes (1 at the second visit, after having received information on the phenomenon during the first visit). The frontalis muscle was often activated contralateral to the abnormal orbicularis oculi (Fig. 1B, C); no patient with orbicularis oculi involvement had simultaneous ipsilateral frontalis contractions. Some complained of associated head (16%) and cervical pain (18.0%).

### Progression and Outcome

The course was stable (53%) or variable (33%), with diurnal fluctuations in one-fifth. Spontaneous remissions were reported in 13 subjects (21%), with recurrence in 2 after 2 weeks and 10 years. Patients with or without remission did not differ with respect to number of visits, follow-up duration, gender distribution, age, occurrence of primary or secondary gain, resolution of pending medico-legal issues and disclosure of the diagnosis of psychogenicity to the patient. At the latest follow-up visit, spasms were present mostly in the lips (84%) and eyelids (51%). Isolated lip involvement was the most frequent pattern (43%), followed by lips and eyelids (23%) and eyelids alone (13%). Platysma was involved in 61% of patients. Extra-facial contractions involved upper limb (30%), neck (16%), leg (16%), and trunk (5%). When present, limb involvement was ipsilateral to the facial involvement. Dystonia was the most frequent phenotype of extra-facial sites (58%), followed by tremor (14%) and other jerks (10%). All but 6 subjects received a variety of medical and nonpharmacological treatments (Supplementary Table 1) without any benefit (56%) or worsening (20%). Aside from complete remissions (following a treatment or not), 20% of treated patients improved after treatment (Botulinum neurotoxin [BoNT] at therapeutic doses was effective in 5 cases, antidepressants in 3, antiepileptics in 2, and psychotherapy in 1).

### Associated Conditions

Depression and headache were common (Supplementary Table 2A). Several features supported a psychogenic etiology (Supplementary Table 2B). Notably, most patients displayed variable phenomenology upon suggestibility maneuvers. A nonphysiologic or placebo maneuver (most often a vibrating tuning fork) improved 16% and worsened 10% of the 19 subjects to whom it was applied.

## Discussion

This is the first series of PMD patients involving the face—exclusively or in association with other extra-facial movement disorders. A common clinical picture emerged, with asymmetric, mostly dystonic involvement of the lower face, resembling oromandibular dystonia, affecting predominantly young women (9:1 female-to-male ratio), and with a variety of features suggesting a psychogenic cause (Table 3).^2^,^6^,^7^,^9^,^11^–^16^ Indeed, the vast majority of these cases received a “positive” diagnosis rather than a diagnosis based on the exclusion of organic diseases.

**TABLE 3:** Features distinguishing organic versus psychogenic oromandibular and facial dystonia

	Organic	Psychogenic
Onset and progression	Gradual, slow progression	Sudden-onset, static course
Sensory tricks	May be present	Rarely present
Most common distribution	Lips	Jaw, eyelids
Most common sidedness	Bilateral	Unilateral
Platysma involvement	Very rare, bilateral	Common, ipsilateral
Orbicularis oculi and frontalis muscle involvement (if present)	Orbicularis and frontalis, ipsilateral	Orbicularis and frontalis, contralateral^a^
Dystonic pattern	Phasic	Tonic
Dystonic exacerbation	Action-induced	Paroxysmal, maximum at rest
Dystonic spread	Segmental to cervical region	Segmental or multifocal
Evolution	Slowly progressive, no spontaneous exacerbations or remissions	Fluctuations in severity, spontaneous exacerbation and remissions
Pain	Usually absent	Present (25%)

a If orbicularis present in isolation, it most often occurred contralateral to the affected lip/jaw.

Involvement of the lower lip with downward deviation at the angle of the mouth combined with ipsilateral platysma co-contraction, previously termed “smirk,”^17^ was the most frequent pattern at presentation. In contrast to the more common involvement of upper facial muscles in organic cranial movement disorders, the involvement of the lower face appears to be characteristic of PFMDs.^18^ Unlike organic oromandibular dystonia, most subjects had asymmetric facial involvement and absence of gestes antagonists. More importantly, the majority of patients had no involvement of speech, which, is commonly seen in oromandibular dystonia and has been reported in other series of PMDs.^19^ Moreover, although focal task-specific dystonias affecting perioral muscles are well described (eg, embouchure dystonia),^20^ “unilateral dystonia of the jaw” is uncommon and was first reported in 1986 by Thompson et al.^1^ in a small series. In retrospect, at least 1 of these patients was subsequently diagnosed as having a psychogenic etiology (J. Obeso, personal communication). Indeed, apart from masticatory spasm, whose clinical features are easily recognizable (eg, the association with facial hemiatrophy),^21^ few references to unilateral jaw spasms are to be found in the literature and several of these have clinical features that might support their reclassification as a PFMD (Table 4).^1,3–5,22^

**TABLE 4:** Patients with unilateral movements of lip/jaw previously reported

Reference (cases)	Age/ sex	Onset/accompanied symptoms/precipitating events	Neurological examination	Natural history/response to treatment	Authors' comments
1(1)	14/F	Pain and tingling in the left mandible; chewing and talking difficulties	Tonic, sustained deviation of jaw to the left	No spreading; improvement with benzhexol	Focal dystonia of the jaw confirmed by: (A) presence of dystonia in other body parts (cases 2, 3, and 4); (B) continuous EMG activity of lateral pterygoid muscle at rest (case 1); (C) abnormalities in the recovery cycle of the blink reflex (case 3); (D) paroxysmal attacks of dystonia similar to those described in MS (case 5: normal imaging studies); and (E) improvement with anticholinergics
1(2)	30/F	Numbness on left side of face; tongue biting during the facial spasms	Torticollis and chin deviation to the left together after two years of forced and painful opening of the mouth	Spreading; partial benefit with benzhexol	
1(3)	35/F	Dental extraction; chewing difficulties and tongue biting during the facial spasms	Sustained deviation of jaw to the right associated with intermittent ipsilateral torticollis	Transitory improvement with benzhexol and with amitriptyline thereafter	
1(4)	22/F	No precipitating events reported	Intermittent left blepharoclonus and sustained deviation of jaw and lip to the left associated with ipsilateral torticollis and arm dystonia in the outstretched position	No relief with anticholinergics, carbamazepine, clonazepam, tetrabenazine	
1(5)	28/F	No precipitating events reported	Right eye closure followed by upward deviation of the right corner of mouth; left eye adduction and spasms of orbicularis oris, mentalis, and left frontalis muscles; flexion and inversion of right foot while walking		
22(1)	52/F	Phantom canine teeth and chronic facial pain after resection of hypertrophic gums	Tonic, sustained upward retraction of right corner of the mouth	No spreading; Improvement with doxepin and oxycodone	Peripheral injury induced dystonia
3(1)	40/F	Abrupt; associated with head and neck pain	Tonic, sustained, lateral and outward protrusion of the right lower lip	No spreading; No response to treatment; spontaneous improvement over time	Though unusual for primary dystonia (worsening at rest and improvement with labial movements) the stereotyped lip movements could not be entirely explained by a psychogenic cause
3(2)	41/F	Abrupt; accompanied by headache and left sided weakness	Tonic, sustained, lateral outward protrusion of the left lower lip		
3(3)	25/F	Abrupt; associated with headache and right sided weakness		Spreading to ipsilateral eyelid; spontaneous improvement over time	
3(4)	42/F	Abrupt; accompanied with headache and left sided weakness		Lost to follow-up	
4(1)	27/F	Abrupt; numbness in right cheek and right half of tongue	Tonic, sustained, lateral and outward protrusion of the right lower lip and right jaw deviation; present during sleep	Improvement with BoNT	Habit spasms superimposed on an abnormal faciotrigeminal motor function after a Bell's palsy
4(2)	39/F	Headache and numbness in right side of face			
5(1)	27/F	Acute onset after facial and trigeminal lesion secondary to a spider bite	Tonic, sustained downward deviation of left lower lip	Long-term improvement with a maxillary splint	Alteration of trigeminal input with secondary unbalanced inhibitory-excitatory activities within basal ganglia circuits

F, female; EMG, electromyogram; MS, multiple sclerosis; BoNT, botulinum neurotoxin.

The unilateral involvement of facial muscles is common to PFMDs and hemifacial spasm (HFS). However, unlike the synchronous myoclonic jerks or tonic contractions,^23^ most patients with PFMDs show asynchronous, generally tonic contractions in ipsilateral lower and upper face or synchronous bilateral contractions of the lower face. In addition, we specifically looked for the “other Babinski sign” described in HFS patients with a specificity of 100% and characterized by ipsilateral eyebrow rising during eye closure due to the simultaneous contraction of orbicularis oculi and the internal part of the frontalis.^24^ This sign was not found in any of the patients with asymmetric spasm of orbicularis oculi, who, rather, had the eyebrow rising contralateral to the closing eye. Psychogenic HFS has been previously reported by Tan and Jankovic^2^ and was recently reviewed in the same center,^25^ where it accounted for 7.4% of all the cases referred for HFS. Almost all patients were women (15/16), mean age at onset and disease duration of symptoms were 37.4 ± 19.5 and 1.7 ± 2.2 years, respectively. Described patients had an acute onset of symptoms, a nonprogressive course, fluctuations in symptom severity, inconsistent signs, findings incongruent with HFS or facial dystonia, spontaneous improvement, and normal diagnostic studies. Facial spasm was characterized by upward or lateral deviation of the corner of the mouth;^2^ with bilateral involvement observed in 7 patients.^25^ Similar to our cases, no patients reported facial spasms during sleep (present in up to 80% of organic HFS)^26^ or worsening of spasms during voluntary facial contractions (documented in up to 39% of HFS patients);^26^ and most patients had lower-face involvement at onset in contrast to the isolated lid involvement typically present at onset in organic HFS. Interestingly, the report of a vascular loop compressing the seventh cranial nerve in 1 of our patients emphasizes the potential for this finding to be present in asymptomatic patients and the need for clinicians to recognize the important differences between HFS and PFMDs in order to avoid unnecessary diagnostic tests or treatments including surgical decompression. Electrophysiological examination may help in distinguishing HFS from other abnormal facial movements by demonstrating ephaptic impulse transmission between different facial nerve branches.^27^ Indeed, a neurophysiological hallmark of HFS is the spread of the blink reflex (BR) responses elicited by supraorbital nerve stimulation to muscles other than the orbicularis oculi. Interestingly, BR responses were normal when tested in 10 of our patients with PFMDS.

An uncommon involvement of the orbicularis oculi was demonstrated by 2 patients who could not open their eyes, a condition resembling “eyelid opening apraxia.”^28^ The strength of patients' eye closure varied depending on the force exerted against the eyelids by the examiner. In some instances, the pattern of abnormal movement was very complex and not falling into any of the better defined movement disorders affecting the face. Examples included left blepharospasm with intermittent bilateral lower eyelid contractions associated with bilateral clonic movements of the levator labii superioris alaeque nasi muscle, or blepharospasm associated with movements of downward pulling of both angles of mouth followed by upward deviation and mouth opening. An important clue was provided by the ipsilateral contraction of the platysma, a muscle uncommonly affected by organic movement disorders with the exception of HFS, tics, and some cases of cranial dystonia, where it is usually bilateral. Interestingly, none of our cases reported oculomotor involvement (ie, oculogyria or convergence spasm), which has been recently reported in 13 PMDs patients, 1 of whom had facial involvement.^29^ Finally, among patients with paroxysmal PFMDs, none acknowledged voluntary control, urge, or relief of urge after the movements, distinguishing these from tics. Moreover, these patients never displayed rapid movements and spasms were more sustained than those observed in dystonic tics.

Fixed dystonia of the oromandibular region has been reported to result from peripheral injury^30^ and may develop within hours to months after dental procedures.^31^ This atypical cranial dystonia exhibits persistent pain and dysesthesia, reminiscent of the limb causalgia syndrome, is also suggestive of a psychogenic etiology.^32^ Fixed dystonic posture, in combination with sensory, autonomic, and motor disturbances, is a well recognized manifestation of complex regional pain syndrome (CRPS), reported as a complication of local trauma, often without peripheral nerve lesions.^32^ Although most cases concern a limb, CRPS with focal dystonia can involve muscles supplied by the cranial nerves as well.^31^ The diagnostic dilemma of dystonia following minor peripheral injury remains a major source of controversy in this field given the psychogenic features exhibited by these patients.^33^ Future electrophysiological studies might help to unravel this controversy as BR recovery cycle^8^ and sensorimotor plasticity^34^ have been found normal in psychogenic blepharospasm and psychogenic limb dystonia in contrast to the organic counterparts. Nevertheless, it should be acknowledged that electrophysiological studies might also disclose abnormal findings in patients with PMDs.^1^,^35^

Albeit commonly reported in PMDs literature,^15^ the large preponderance of female gender in our sample is in keeping with another series of PFMDs patients,^25^ thus suggesting that women are particularly prone to develop facial involvement in a context of psychogenicity. Finally, in our sample, depression is the most common associated condition with a prevalence by far higher than observed in the same age group of women in the general population (38% vs 4%^36^). By contrast, although very common, the prevalence of tension-type headache is not higher than reported in the same population without PFMDs.^37^

Our study has several limitations. The major shortcoming is represented by its multicenter retrospective design, which is open to several errors (eg, missing data, ascertainment errors, nonuniform modes of assessment, recording data, and follow-up). However, since the facial phenotype of PMDs has not been fully characterized, its study could only arise from retrospective review rather than a prospective design. The reliance on 7 centers employing the same standardized data collection was intended to increase the reliability of ascertainment of this rare phenotype and increase the generalizability of the reported observations. Moreover, the diagnosis of PMDs has been challenging because of historic transitions through the pitfalls of underrecognition, misdiagnosis, overdiagnosis, and diagnosis by exclusion.^38^ Furthermore, in the United States, the management of PMD patients is hampered by poor physician reimbursement, future insurability of patients, and ongoing litigation.^39^ Although most PMDs (and also organic disorders such as Parkinson's disease), have no definitive “test” or “biomarker,” the diagnosis can be reliably made by judicious application of diagnostic clinical criteria. Thus, it has been repeatedly emphasized in recent literature that this is not a diagnosis of exclusion but that positive features are critical in making a diagnosis of a definite psychogenic movement disorder.^19^ While in the past, cases of organic dystonia have often been mischaracterized as psychogenic, many of us are impressed that the reverse situation probably occurs more often now, with psychogenic dystonia misdiagnosed as organic. Our report is not only the first case series of psychogenic cranial dystonia (and other movements) but also the first to recognize as probably psychogenic some cases of disorders affecting the oromandibular region previously reported as organic, as discussed above. Despite this large series of a relatively rare disorder (61 patients collected by 7 tertiary-referral centers), it is possible that the phenotypic range may still be larger than acknowledged and biases of ascertainment and referral pattern may have affected the results. Finally, the exclusion of patients with organic movement disorders, precluded our ability to address the coexistence of organic and PMDs, commonly seen in many patients.^19^

In summary, PFMD should be considered when a patient exhibits any combination of the following features: (1) fixed or paroxysmal unilateral facial contractions, specially with lower lip with or without ipsilateral jaw involvement, of maximal severity at onset; (2) inconsistent features such as changes in side and pattern during or between examination; (3) associated somatizations or nonphysiologic sensory or motor findings; (4) reduction or abolition of facial spasm with distraction; (5) response to suggestion or psychotherapy; (6) rapid onset and/or spontaneous remissions; and (7) normal neurological examination. Supportive features are young age, female gender, and associated medical conditions such as depression, headaches, facial pain, fibromyalgia, or irritable bowel syndrome. A prompt diagnosis based on phenomenology will avoid the extensive diagnostic workup characteristic of a diagnosis-of-exclusion approach, prevent unnecessary costly investigations, and permit the institution of appropriate physical, psychological, and medical therapy.^9^

## Legends to the Video

**Video 1**. This patient had sudden-onset right-arm weakness and fixed tonic muscular contraction affecting the right lower lip. Lip contraction attenuated when opening the mouth and protruding her tongue, and disappeared when laughing or trying to whistle, which functioned as a distracting maneuver. A “la belle indifference” approach is noted. The expression at rest with improvement or disappearance with activity (“paradoxical dystonia”) is opposite to the typical action-induced expression of focal dystonias.

**Video 2**. Segment 1. Patient seen 5 months after sudden onset of fixed jaw deviation. Segment 2. Evaluation one month later, after sudden onset of contralateral blepharospasm. Distractibility with tongue movements and variability to passive manipulation of jaw opening and closing are demonstrated. Segment 3. Evaluation 1 year later demonstrates resolution of the blepharospasm but persistence of the fixed jaw dystonia.

## Supplementary material

Supplementary Table 1. Treatments used in patients with PFMDs enrolled in this series. These treatments were generally ineffective as an improvement was reported in only 20% of treated patients (see main text for details).

Supplementary Table 2. Medical conditions associated and features supporting a psychogenic etiology in the present cohort of PFMDs patients.
