# Supplementary material for: Psychogenic Facial Movement Disorders: Clinical Features and Associated Conditions
Source: Mov Disord. 2012 Oct 2;27(12):1544–51. doi: 10.1002/mds.25190 (PMC3633239; doi:10.1002/mds.25190)
Supplement: Supplementary file 1 [file mds0027-1544-SD1.doc]

Supplementary Table 1. Treatments used in patients with PFMDs enrolled in this series. These treatments were generally ineffective as an improvement was reported in only 20% of treated patients (see main text for details).

| *Non-pharmacological* | *N=* | *Pharmacological* | *N=* |
| --- | --- | --- | --- |
| Psychotherapy | 5 | Antidepressant | 15 |
| Cognitive behavioral therapy | 2 | Botulinum neurotoxin | 14 |
| Hypnosis | 1 | Benzodiazepines | 9 |
| Acupuncture | 1 | Anticholinergic | 2 |
| Other* | 6 | Neuroleptics** | 1 |
| None | 38 | Other**^†^** | 20 |
|  |  | None | 6 |

*: Including: physiotherapy, massage, chiropractic treatment. **: prescribed after PFMD onset. **^†^**: including antiepileptics, baclofen, and morphine derivate.
