# Supplementary material for: Psychogenic Facial Movement Disorders: Clinical Features and Associated Conditions
Source: Mov Disord. 2012 Oct 2;27(12):1544–51. doi: 10.1002/mds.25190 (PMC3633239; doi:10.1002/mds.25190)
Supplement: Supplementary file 2 [file mds0027-1544-SD2.doc]

Supplementary Table 2. Medical conditions associated and features supporting a psychogenic etiology in the present cohort of PFMDs patients.

| *A) Medical condition* associated with PFMDs in our cohort^a^ | *%* |
| --- | --- |
| Depression | 38.0 |
| Tension headache | 26.4 |
| Migraine | 25.9 |
| Anxiety | 18.0 |
| Fatigue | 17.6 |
| Fibromyalgia | 9.8 |
| Hypertension | 4.9 |
| Temporo-mandibular joint dysfunction | 3.9 |
| Irritable bowel syndrome | 3.8 |
| Hearing loss | 3.7 |
| *B) Feature supporting a psychogenic etiology* | *%* |
| Historical information: |  |
| Employed in allied health professions | 28.0 |
| History of minor trauma | 26.8 |
| Exposure to a disease model | 18.5 |
| History of physical abuse | 4.3 |
| History of sexual abuse | 2.1 |
| Clinical course: |  |
| Rapid onset | 96.7 |
| Non-progressive course | 85.2 |
| Remissions | 21.3 |
| Suggestibility: |  |
| Movements decrease with distraction | 89.6 |
| Placebo effect­­^b^ | 89.5 |
| Movements increase with attention | 86.0 |
| Resolution when the patient feels unobserved | 61.1 |
| Ability to trigger or relieve the abnormal movements^c^ | 36.4 |
| Disability: |  |
| Functional disability out of proportion to exam findings | 47.2 |
| Selective disability^d^ | 28.1 |
| Secondary gain^e^ | 20.3 |
| Accompanying features: |  |
| Other somatizations^b^ | 49.2 |
| False sensory complaints^f^ | 34.4 |
| Deliberate slowness of movements | 27.9 |
| False (give-away) weakness^b^ | 18.0 |
| Delayed and excessive startle response to a stimulus | 1.6 |
| Self-inflicted injuries | 0.0 |

# ^a^: other, less common (1 case each), conditions were: breast cancer, hypothyroidism, ovary dermoid cyst, othosclerosis, miscarriage, spina bifida oculta, thoracic outlet syndrome, cervical cancer treated with radiation, gastroesophageal reflux disease, osteoarthritis, morbid obesity, intestinal malabsorption; ^b^: see text for details; ^c^: by using nonphysiological interventions (e.g. trigger points on the body, tuning fork); ^d^: defined as disability limited only to specific activities of daily living ^e^: defined as ongoing or pending litigation, disability benefits, release from personal/legal/social/employment responsibilities, and/or increased personal attention; ^f^: e.g. blurred vision, pain, numbness or sense of swelling not following anatomy (whole or half body, ipsilateral hand and foot).
